# Selections

**Published:** 1872-02

**Authors:** 


### Selections.

*Sclerosis Uteri: One of the Sequelæ of Puerperal Metritis.* A Clinical Lecture, by Prof. ALEXANDER J. C. SKENE, M.D., at the Long Island College Hospital, Brooklyn, N. Y. (Reported by E. S. Bunker, M.D.)

GENTLEMEN — You will readily call to mind the patient who had endometritis, giving rise to menorrhagia, and I trust you also remember the clinical fact, then so strenuously insisted upon, namely, that menorrhagia, not accounted for by any other conditions, is a common and most valuable symptom of endometritis.

In direct and striking contrast with that case, I present you one to-day, not of such frequent occurrence as the former, but presenting, nevertheless, points of extreme interest in its pathology and clinical history.

This patient informs us that she is 35 years old, has been pregnant five times, and has given birth to four living children. While pregnant at the seventh month, with her fourth child, she received an injury which caused her to give birth to a dead fœtus a few days afterwards.

During her fifth pregnancy, four years ago, she received a shock from seeing a friend in a convulsion; labor came on immediately, and she was delivered of a seven months' child.

Soon after her confinement she complained of pain and tenderness in the region of the uterus, followed by fever and delirium. These symptoms extended over a period of three weeks, and there can be little doubt, from the history given, that she had acute puerperal metritis, which left her health permanently impaired. Since that time her menses have been irregular, scanty, and attended with pain. At times she has a menstrual *molimen*, but no catamenial flow. During the last year she has menstruated only twice, the last time three months ago. So much for the previous history of the case.

She now suffers from extreme debility and anæmia, as you can see by her general appearance, and complains of ill-defined aching pains throughout the pelvis, but more especially in the sacral region; and occasionally she has very slight leucorrhœa. Her digestive organs are also very much deranged, and her nervous system, from the joint action of disease and drugs, is a miserable wreck.

By physical exploration I find that the uterus is enlarged, being an inch larger than normal. The body and cervix are tender to the touch, and the sound, carried into the cavity, gives extreme pain. The cervix is indurated and smooth, and the os is smaller and more circular than is usually found in those who have borne children.

Exploring the cavity with the sound, I find that while the long diameter is considerably increased, the antero-posterior and lateral diameters are relatively, indeed absolutely, shortened. The uterine walls appear to lie in close contiguity, so that it is impossible to turn the sound far in any direction.

These signs obtained by the probe are of vast importance, for they indicate clearly that the enlargement of the uterus is due to an actual increase in the *walls* of the organ, and not to a mere expansion of its cavity, as in the case of endometritis, which I showed you the other day. In other words, the growth is concentric, not eccentric.

The cervix, as seen through the speculum, is notably pale; the os is small, with its lips curved *inward.* This retraction, or drawing inwards of the os, is confirmatory of the opinion that the walls of the cervix are enlarged more than the mucous membrane of the cavity. When the mucous membrane of the cervix is swollen, and the walls remain normal, the lips are enlarged or pouting.

Briefly, then, the physical signs indicate that there exists a condition of unusual *hardness* and *enlargement* of the uterine walls, while the relative or even actual size of the cavity is lessened. The uterus is also anæmic, as you know from a glance at the cervix. And, before closing the history, let me attract your attention to the fact that our patient has amenorrhœa—a condition that is much more common among the young than with those who have borne children.

Now you cannot fail to have observed that this case presents many points of resemblance to that of endometritis, which occupied your attention at our last lecture. It is on account of these resemblances that I present you this case to-day. By placing them in juxtaposition, as it were, when the subject is still fresh in your memory, you will be better prepared to study the points of difference; for both the past history and present condition show that the cases are essentially different.

This patient's trouble began with acute inflammation of the uterus; the other's did not. This one had amenorrhœa; the other had menorrhagia. In the case before us the uterine walls are enlarged and the cavity diminished, the reverse almost of what obtained in the other. The uterus, in this case, is indurated and anæmic; in the other, it was relaxed and highly congested. These are plain outline distinctions, easily recognized at the bedside, and characteristic of almost opposite pathological conditions.

Excluding, then, the only uterine disease that might lead us into error, and basing our opinion on the clinical history and physical signs, we are compelled to pronounce this a case of what is usually known and described as "*chronic metritis.*"

But I fear the term "*metritis*" will lead you astray. It certainly does not give you a true idea of the pathology of the disease—at least, if you recognize inflammation only by the presence of hyperæmia, the exudation of lymph, suppuration, etc. In this case we have no such evidence of the inflammatory process; the present condition is not that of an inflammatory disease. It bears no more resemblance to ordinary inflammation than the stricture of the male urethra does to an acute gonorrhœa. Nor is the condition that of induration the result of the inflammatory process. That usually results in the *contraction* as well as hardening of the tissue in which fibrin is effused. Now, in the case before us, you will observe two conditions not often associated except in malignant disease, namely, hardening, with *increased size* of tissue. Clinically considered, this condition is not unfrequently observed associated with a history and symptoms such as we gather from this patient, and in the absence of any name to indicate the condition, I venture to suggest that of *sclerosis*—a term already familiar to you as designating morbid conditions of other organs similarly affected.

I confess to you my dislike to introducing new terms into the literature of gynæcology, and certainly would not if I did not think the one usually employed, namely, "chronic metritis," gives no true idea of the nature of the lesion.

The term *sclerosis* simply means hardening, and when applied to the inter-cellular connective tissue, it includes also thickening or growth of this structure. It resembles the condition described by Virchow as "nutritive irritation of connective tissue." Your knowledge of anatomy will enable you to estimate the relations of this tissue to the varied structures of the body. In its normal state it is an exceedingly delicate structure, and is extensively diffused in all parts of the body. It is the bond of union to the framework of the body as the plaster is to the bricks; it is the delicate curtain upon which the minute capillaries are spread; and it follows, of course, that the increased growth of this connective tissue takes place at the expense of the fibro-muscular tissue of the uterus, a condition of local anæmia is produced by pressure on the capillary vessels, and I need scarcely add that function and force are correspondingly destroyed. This lesion has been specially studied of late as it occurs in nerve-centres. Hypertrophy of the "neuroglia," or connective tissue in *patches*, gives rise to a condition resembling *locomotor ataxia*.

Now the result of this same proliferation of the connective tissue cells, from some previous spur of excitement, is precisely what we find in this uterus. We have the " nutritive irritation " of Virchow, affecting the connective tissue cells at the expense of the other structures of the part, and not simple induration from previous inflammation. The organ is rendered abnormal in size, dense in structure, brittle in quality, and is anæmic or bloodless.

In the older pathology we have no word that represents this condition. We can scarcely believe that the process is merely one of exudation of lymph into the tissues of the uterus, for that is usually followed by more or less *contraction;* nor can we for a moment believe that there is excessive cell-growth from malignant tendency. Thanks to our German brethren, they have pointed out and accurately described the lesion, and applied to it the name of "*sclerosis*." And no other term, in my judgment, so perfectly represents a certain class of uterine diseases, a typical case of which is now before you.

I would not be understood, however, as denying that there is inflammatory exudation. On the contrary, there is every reason to suppose, from the natural history of the disease, that there is more or less effusion of inflammatory lymph into the tissues of the uterus. But the hypertrophy of the cellular tissue, and consequent *enlargement* of the organ, are so much more prominent characteristics of the disease, that I have selected the term *sclerosis*, rather than metritis, to describe it.

It would be interesting in this connection to study how the lesions of sclerosis uteri are developed in their natural order of sequence; but time will not permit at present. Let me refer you for information on this point to a most admirable article on "*Chronic Metritis in its Relations to Malignant Diseases of the Uterus*," by Dr. Noeggerath, of New York. You will find it in the *American Journal of Obstetrics* for November, 1869. I will read you a brief extract:—

"The disease in question is the combined result of sub-involution of the uterus, and a process of exudation which takes place in the tissues of the uterus immediately after delivery, in consequence of puerperal fever. This interruption of retrogressive metamorphosis takes place whenever the natural involution of the uterus is interfered with by some injurious influences, so that the absorption of the exuded mass deposited between the muscular elements cannot be properly accomplished. This results in the development and permanent deposit of cellular tissue within the walls of the uterus."

Just here there comes up a question to which I can give but a single remark at present: Does sclerosis uteri arise exclusively from puerperal metritis? Or may it be developed from other morbid influences? Dr. Noeggerath says he has never seen the disease occur as a sequel of any other affection. My own experience accords with his. We should naturally suppose, however, that long-continued hyperæmia and nervous irritation might favor the proliferation of the connective tissue cells in the uterine walls, as well as elsewhere; but we have no proof as yet that sclerosis uteri is developed in that way.

Finally, sclerosis uteri is a *permanent* lesion. There is no tendency to restoration of normal tissue; on the contrary, there is lowered vitality of the part, and consequent tendency to still further degeneration. The affection exemplifies the general pathological law, to which there is no exception, that whenever the tissues of an organ are changed from their normal type, that change is towards degeneration, decay, and death. Hence the practical fact that these cases of sclerosis are peculiarly prone to such diseases as epithelioma, *cauliflower excrescence* of the cervix, etc., etc.

Malignant disease of the uterus can often be traced back through a natural history such as I have pointed out in this case; and that the tendency to malignant action is hastened in such cases by the injudicious use of caustics, I have no doubt. It is important, therefore, that we shall early recognize the difficulty, and avoid a course of treatment that must end, sooner or later, disastrously to the patient.

Trusting that you comprehend the pathology of sclerosis uteri and its relation to other morbid states, I will now briefly recapitulate some of the leading points in the diagnosis of this affection.

1. A clinical history indicating that the patient has previously had puerperal metritis, followed by an impaired state of health.

2. Constitutional symptoms such as occur in chronic forms of uterine disease.

3. Derangement of function: usually irregular, scanty, and painful menstruation. The pain continues, as a rule, through the whole period of menstruation, and may be likened to an exaggeration of the ill feelings of an ordinary menstruation. By these peculiarities it may be distinguished from the dysmenorrhœa caused by flexion of the uterus. In this latter affection the pain is that of intense uterine colic, and is usually limited to the beginning of menstruation, decreasing or disappearing when the flow begins.

4. Enlargement and induration of the uterine walls.

5. Increased length of the uterine cavity, without increase of the lateral and antero-posterior diameters.

6. Anæmia of the uterus, indicated by the amenorrhœa, and the pale appearance of the cervix.

7. Absence of cervical disease, such as occurs in hyperæmic and inflammatory affections.

8. The slight retraction of the lips of the os, and the small diameter of the cervical canal in proportion to the increased size of the cervix.

---

*On the Treatment of Purulent Ophthalmia in New-born Children.* By R. Liebreich, Ophthalmic Surgeon and Lecturer to St. Thomas's Hospital, London.

Gentlemen—This is not a disease which is amenable to treatment only up to a certain degree, and which may lead to unfavorable results even under the most rational treatment; but, on the contrary, it is an affection which, even in its worst form, is always perfectly curable.

What, now, is this treatment? It consists, first, in careful cleansing; secondly, in the local application of cold; and, thirdly, in cauterizations with mitigated nitrate of silver (one part of nitrate of silver and two parts of nitrate of potassium fused together). Allow me to speak of this in a somewhat elementary and detailed manner.

As regards cleansing, it is above all necessary to explain to the attendants the importance of it, for, ordinarily, the fear of injuring the child prevents them from properly opening the eyes in order to remove the secretion. I do not recommend syringes, so generally used, for cleansing—first, because they are dangerous to the attendants, who, in using them, may easily have some of the contagious matter spattered into their eyes; secondly, because by this method the secretion is not completely removed, even after pouring much water over the child. A fine sponge, if you can rely upon its being kept clean, or, if not, small pieces of moistened linen rag, are preferable for effectually cleansing the conjunctiva.

The application of cold, if made in a careful and suitable manner, is of great assistance in the treatment. In the mildest form of the disease this application alone may even effect a cure in a few days. It is then only necessary to apply, for several hours a day, small linen rags, moistened by being dipped in cold water, changing them constantly. In the more serious cases, on the contrary, when there is much swelling, redness and heat in the eyelids, and a copious discharge of thick, yellow, purulent secretion, it is necessary to apply, day and night, without intermission, small rags, previously placed upon ice, and to renew them continually. Later, when the elevated temperature begins to fall, the applications may be discontinued during the night, and gradually reduced, according to the course of the disease. In order to prevent the child from taking cold, it is necessary that the rags should be of a size merely to cover the eyelids without touching the bridge of the nose, and not to make them too wet.

We now come to the real curative treatment—that is, the cauterization. It should only be done with mitigated nitrate of silver. The eyelids should be reversed one after the other, and, after being carefully cleansed, touched with the caustic, which must be passed over all the swollen and red part of the mucous membrane. Before replacing the eyelids in their natural position, it is necessary to neutralize the free nitrate of silver by a drop of salt water. For the first few days only of the disease we may restrict the treatment to the application of cold, and then commence the cauterizations; repeat them once a day, never more frequently, and, after an evident reduction of the disease, once every two or three days. It is important not to repeat the cauterization until the scar of the previous one has disappeared.

To drop weak solutions of nitrate of silver into the eye is not advisable, even in the mildest forms; for the graver forms it is completely insufficient. Cauterization with pure nitrate of silver ought never to be used, neither in this nor in any other disease of the conjunctiva, as it is impossible to limit its effects. The slightest touch with pure nitrate of silver, in fact, produces a strong cauterization, not only limited to the surface of the mucous membrane, but attacking the subjacent connective tissue. The cicatrization which is the result of such cauterizations produces a permanent irritation of the eye, which cannot be removed by any possible means. None of the other known caustics can replace the mitigated nitrate of silver in the treatment of purulent ophthalmia. Let nobody avoid the trouble of preparing a pencil of nitrate of silver himself if he has not a suitable one already prepared at his disposal. The sole difficulty lies in procuring an iron mould, into which a mixture of one part of nitrate of silver and two parts of nitrate of potash is to be poured, after having been melted over the fire. This difficulty could be avoided by immersing an iron wire repeatedly with one end into the melted mass until, on cooling, a sufficiently thick layer of caustic adheres to it. This is the procedure which I formerly recommended for caustic probes in cases in which it is desirable to cauterize with a very fine point or in a very narrow canal (lachrymal canals, lachrymal sac, ciliary roots, etc.).

In the most difficult cases, cleanliness, cold and cauterization are sufficient, and enable you to form a good prognosis. The case is, however, different when children come under your treatment after a more or less great part of the cornea has been destroyed, the iris projecting, the capsule of the lens injured; here the prognosis depends entirely, in the individual case, on the existing destructions. Ulcers of the cornea of no great extent, and not too near to perforation, allow of a still favorable prognosis; but it becomes more unfavorable already, when perforation has taken place, and the iris projects. There is a decidedly bad prognosis if almost the greater part of the cornea had been destroyed before the child came under your treatment.—*London Medical Times and Gazette.*

---

*Clinical Lecture on two Cases Illustrating the Points of Difference between Acute Phthisis and Acute Tuberculosis.* By JAMES H. HUTCHINSON, M.D., President of the Pathological Society of Philadelphia; one of the Attending Physicians to the Pennsylvania Hospital.

The two cases which have suggested this lecture were under my care in the men's medical ward of the Pennsylvania Hospital last year. They both ran an acute course and presented many points of similarity with each other; but there were also points in which they were unlike, and these enabled me to recognize in one instance the presence of acute phthisis, and in the other, of acute tuberculosis. A post-mortem examination in each case fully confirmed the diagnosis. There are many able physicians who refuse to admit the existence of any material difference between these two diseases; but surely to separate two conditions so readily distinguished during life, and characterized after death by such different lesions, ought not to be looked upon as an over-refinement in diagnosis.

Acute phthisis I believe to depend upon a rapid degeneration of the products of a low form of inflammation of the lungs. The origin of acute tuberculosis is not quite so well understood.

The cases are reported briefly in abstract, in order that the diagnostic symptoms in each may be prominently brought out.

CASE I.—D. McG., æt. 18, born in Philadelphia, was admitted into the medical wards of the Pennsylvania Hospital on the 13th of March, 1871. His prominent symptoms at that time were, emaciation, fever, subsultus tendinum, carphologia, muttering delirium during the daytime, occasionally violent delirium at night, and dry and smooth tongue. There were no tympany, no diarhœa, and no rose-colored spots on the abdomen. It was not until the 18th that I learned that the patient had no hereditary tendency to phthisis, that his illness began as bronchitis about five weeks before he came under my care, but that it was not until March 4, that he had been obliged to give up work and go to bed. Upon examining his chest I found that there was dullness posteriorly over both sides, but most marked over the left side, where there was also bronchial respiration on a level with the spine of the scapula. On the 20th he was free from delirium, and able to corroborate the history just given. At this time the abdomen was noted as being scaphoid in shape, and covered with sudamina. The tongue was clean and moist, but fissured and red. There was slight diarrhœa. The physical signs continued as before. On the 23rd there was an improvement in all the symptoms ; no elastic tissue could be detected in the sputa.

On the 24th the patient was not quite so well. A careful physical examination of the chest showed that there was dullness over the whole of the left side of the chest posteriorly, and in a less degree in front down to the third rib, and that bronchial respiration and moist crackling were heard in the superior scapular and the infra-clavicular regions. The patient suffered from profuse sweats. The symptoms increased in severity, the physical signs indicating extensive destruction of the left lung, and commencing disease at the apex of the right lung, until his death, which occurred on the 14th of April.

The autopsy showed that the physical signs had been correctly interpreted ; caseous degeneration and excavations being found in both lungs.

The following is the record made of the pulse, respiration and temperature during a portion of the time he was under treatment :

| | A. M. | | | P. M. | | |
|---|---|---|---|---|---|---|
| | Pulse. | Resp. | Temp. | Pulse. | Resp. | Temp. |
| March 13 | | | | 126 | 36 | 104 |
| " 14 | 106 | 28 | 100½ | 118 | 38 | 104 |
| " 15 | 118 | 28 | 100 | 112 | 34 | 102 |
| " 16 | 104 | 38 | 100 | 112 | 34 | 102½ |
| " 17 | 104 | 28 | 99 | 116 | 40 | 102 |
| " 18 | 112 | 38 | 101 | 118 | 44 | 102 |
| " 19 | | | | 120 | 44 | 102 |
| " 20 | 126 | 46 | 101 | 120 | 40 | 102½ |
| " 30 | | | | 130 | 58 | 103 |
| " 31 | 120 | 40 | 101 | 136 | 36 | 101 |
| April 1 | 120 | 30 | 99 | 134 | 26 | 102 |
| " 2 | | | | 135 | 40 | 102 |
| " 3 | 120 | 40 | 100 | 130 | 44 | 101 |

Case II.—J. B., æt. 25, single, born in Ireland, puddler, was admitted into the medical wards of the Pennsylvania Hospital April 13, 1871.

His mother died of typhus fever, aged 30 years. His father is still living, in good health. No history of phthisis in any branch of his family could be discovered. He stated that his health had always been perfectly good up to the beginning of March, 1871, when through exposure incident to his occupation he caught what he described as a severe cold. This was accompanied by sharp pain in his chest, much shortness of breath, and fever, but by little cough or expectoration. From this condition he recovered sufficiently to resume his former occupation. Three weeks later, while still suffering slightly from this attack, his cough increased in severity and frequency ; his expectoration occasionally contained streaks of blood, and very rarely consisted of blood alone, but at no time was there a large amount spit up. He also stated that he had had a chancre ; but there was no reason to believe that he had ever suffered from secondary syphilis. When admitted, his appetite was good, his bowels were regular, his urine was acid and had a specific gravity of 1024 ; it contained no albumen and no sugar. He was suffering from a slight cough and some mucous expectoration. The physical signs were as follows : some dullness on percussion over the lower part of the left lung posteriorly. The respiratory murmur on both sides was harsh and somewhat blowing ; the expiratory murmur was prolonged. Sonorous and sibilant rales were heard in the interscapular region. A loud blowing systolic murmur was heard near the ensiform cartilage. On the 30th of April the physical signs were scarcely more distinctive than on admission, the dyspnœa was extreme, and there was evidence of deficient aeration of the blood in the blueness of the lips, nose, and finger-ends.

The diagnosis was acute tuberculosis of the lungs.

Appended is the record of his temperature, pulse, and respiration, as taken from the time of his admission until his death, which occurred May 10, after he had passed from under my care into that of my colleague, Professor J. A. Meigs, who permits me to make the following extract from the post-mortem book:

| | A. M. | | | P. M. | | |
|---|---|---|---|---|---|---|
| | Pulse. | Resp. | Temp. | Pulse. | Resp. | Temp. |
| April 17 | 92 | 24 | 98 | 104 | 30 | 102⅕ |
| " 18 | 104 | 24 | 100 | 98 | 26 | 98 |
| " 19 | 96 | 24 | 100 | 90 | 24 | 99 |
| " 20 | 104 | 26 | 98½ | 92 | 24 | 101 |
| " 21 | 102 | 28 | 101 | 96 | 26 | 99 |
| " 22 | 106 | 28 | 101 | 97 | 24 | 98 |
| " 23 | 104 | 26 | 100 | | | |
| " 24 | 110 | 30 | 102 | 100 | 20 | 98 |
| " 25 | 110 | 30 | 101 | 90 | 26 | 98 |
| " 26 | 104 | 30 | 102 | 94 | 30 | 100 |
| " 30 | 98 | 26 | 99 | 114 | 32 | 103 |
| May 1 | 104 | 32 | 100 | 110 | 34 | 101½ |
| " 2 | 112 | 30 | 99 | 106 | 28 | 100 |
| " 3 | 104 | 30 | 100 | 104 | 30 | 100 |
| " 4 | | | | 106 | 30 | 100 |

"Autopsy twelve hours after death. Rigor mortis well established. Emaciation not marked. *Thorax.*—Abundant pleuritic effusion in right cavity, also a few recent adhesions; right pulmonary pleura thickened. Left pleural surfaces tightly adherent throughout a considerable portion of their extent, mostly at apex and posteriorly. Thick false membrane over the remainder of the pulmonary pleura. Sac contained serum, mingled with blood and pus.

"The whole of both lungs studded with miliary tubercles; abundant purulent exudation escaped on section.

"No cavities were discovered.

"*Heart.*—Right auricle dilated; bicuspid valve insufficient. Walls of left heart increased in thickness.

"*Liver* congested.

"*Spleen* enlarged and friable.

"Glands in the anterior mediastinum enlarged and indurated; no cheesy appearance."

There can be no doubt, both from a consideration of the post-mortem appearance and of the physical signs presented by the patient when I last examined him, that the right-sided pleurisy was set up after he passed from under my care. The pleurisy on the left side, on the contrary, had doubtless been the cause of his first illness, and possibly indirectly of the fatal one; for the pus in the pleural sac was evidently old and thickened, and the serum and blood with which it was mingled had in all probability been extravasated only a short time before death.

As I have said, the cases resemble each other in some points and differ in others. Let us compare them. They both ran an acute course, each coming under my care soon after the commencement of the morbid process, and remaining in the wards of the Pennsylvania Hospital about a month, when death took place. Both patients presented some of the general symptoms of disease of the lungs; in both the respiration was frequent, the temperature elevated, and the pulse accelerated; both had had an illness prior to that for which they sought admission to the hospital. But here the resemblance ceases. In the first case, the symptoms at first suggested to all who saw the patient the impression that he was suffering from typhoid fever, but upon closer examination many of the characteristic symptoms of that disease were found to be absent. The history indicated bronchitis, or possibly catarrhal pneumonia, as the starting-point of the disease. Very soon after his admission, evidences of consolidation of parts of the lungs were detected by the physical methods of diagnosis, and about ten days later crackling rales could be heard in positions where bronchial respiration and dullness had been discovered. But during the whole of this patient's illness there was never more interference with the respiration than could be satisfactorily accounted for by the physical signs. Moreover, during the last three weeks of his life all the general symptoms could be referred to the disease of the lungs which was known to exist. The temperature-record shows that there were marked evening exacerbations, the thermometer frequently recording a temperature in the evening three degrees higher than in the morning. This is, of course, always the case in any disease in which hectic occurs. The sweating was also a marked feature of the case, and was quite as profuse as we generally see it in cases of chronic phthisis. The sputa, too, a short time after admission, were of the character usually found in cases in which rapid destruction of the lung is taking place.

In the second case, the pleurisy, which had occurred a few weeks before the patient's admission to the hospital, had left traces which were distinctly recognized when he first came under my care. In this case there were symptoms which the physical signs did not explain; for these, it will be remembered, were at no time, when taken by themselves, indicative of serious disease of the lungs. Thus, the dyspnœa was extreme, there was cyanosis of the finger-ends and of the lips, while upon percussion the dullness due to the previous pleurisy could only be detected, and auscultation revealed the presence merely of harsh respiration, sonorous and sibilant rales, and some prolongation of the expiratory murmur. Cough was a constant symptom, but it was dry and hacking, or was accompanied by the expectoration of a small quantity of mucus, which was sometimes, but not often, streaked with blood. Sweating very rarely occurred.

The temperature noted in this case was high, but not so high as the preceding, and the diurnal range was also marked—much more marked than is usual in cases of acute tuberculosis. But, whereas in the former case the exacerbation occurred in the evening, in this case it occurred in the morning; and you will find that this sometimes happens when tuberculosis has supervened upon phthisis, as well as when it is, as in this case, an original affection, or, to speak more correctly, has not been preceded by any disease of the lungs.

The existence of a systolic murmur at the ensiform cartilage, together with the increased area of percussion-dullness in the region of the right ventricle, indicated that the right ventricle was dilated —a condition which it seems to me must almost of necessity occur in consequence of the stasis of blood which results from the interference with the circulation which a large number of miliary tubercles in the lungs must exert. The blueness of the lips and finger-ends undoubtedly arose from this venous congestion; and it was difficult to explain this in any other way, since there was no history of previous disease of the heart.

It was impossible to convince all who saw the patient with me that he was really the subject of so serious a disease as acute tuberculosis. There was in this case no marked prostration, sweating, or profuse expectoration, as in the other case, which, taken together with the absence of the physical signs of disease of the lungs, seemed to the minds of some to render untenable the diagnosis I had made.

I have on a former occasion endeavored to explain to you in what way phthisis is produced, and I intend in this lecture to attempt to give you some idea of the pathology of tuberculosis. Niemeyer, in his "Clinical Lectures on Pulmonary Phthisis," says that "tuberculosis, in most cases, is a secondary disease, arising in a manner not known to us through the influence of caseous morbid products on the organism."* These caseous morbid products are more usually found in the lung, but in some cases of tuberculosis, as in the one under consideration, we have to look for them elsewhere. In such cases we shall find the glands diseased, or perhaps an inflammatory exudation on the pleura, the peritoneum, or elsewhere, presenting evidence of caseous degeneration. Otitis, caries of the bones, and other affections, accompanied by much suppuration, are said in some instances to have given rise to it.

It is difficult to understand in what way these caseous products within the body excite the deposition of miliary tubercles in the various organs of the body. Tuberculosis has been artificially produced in the body of the lower animals by the inoculation of various substances, which, however, have always this in common, —that they contain corpuscular elements; and, provided these be not destroyed, the substance to be inoculated may be acted

* Parke's Translation.

upon by various chemical agents without impairing its power to produce the disease. If tuberculosis may be caused by the introduction of corpuscular elements from without, there can be no reasonable objection to the acceptance of the hypothesis that this *infection* or inoculation may sometimes be due to the absorption of corpuscular elements contained within the body, it matters not in what pathological process they may have originated. These, when absorbed, act as foreign bodies, and cause within the blood-vessels a derangement of the circulation, which may in some instances amount to partial stasis. As a consequence of this, there is an extravasation of a number of the white corpuscles of the blood, which generally carry with them these foreign bodies, and, collecting together in a mass, form the basis of a tubercle, which is completed by the growth of a capsule of connective tissue around it.

We learn from the record of the post-mortem examination in J. B.'s case that the left pleural cavity contained thickened and caseous exudation, and I think we must trace the occurrence of acute tuberculosis to absorption of some of the corpuscular elements it contained. It is difficult to understand why tuberculosis is set up in only a small proportion of the cases in which the predisposing cause is present. Perhaps many phthisical patients escape tuberculosis in consequence of the protection afforded against absorption by the increased growth of connective tissue, which often forms a capsule around the caseous matter in the lungs. This view is sustained by the fact that, as was known to Laennec, a deposition of miliary tubercles often occurs a short time before death, when by reason of the softening and destruction of the lung, which is very apt to occur in the last stages of the disease, the protecting barrier may be supposed to be broken down.

The attempts to produce tuberculosis in the lower animals are not uniformly successful, and it is therefore not unreasonable to suppose that the cases in which everything seems to be favorable to it, and which yet escape the infection, do so in consequence of the absence of an unknown but important factor.—*Philad. Medical Times.*

---

*On the Treatment of Hemorrhoids in Pregnant and Puerperal Women.* By FORDYCE BARKER, M.D., Professor of Clinical Midwifery and Diseases of Women in the Bellevue Hospital Medical College, Obstetric Physician to Bellevue Hospital, etc.

While all who are engaged largely in obstetric practice must have encountered a great deal of suffering from hemorrhoids in pregnant and puerperal women, the young practitioner, who consults his books for directions as regards the management and treatment of this painful affection, will find in his text-books on midwifery and diseases of women only the most meager and unsatisfactory allusions to it. Many of these works make no allusion to this subject, and in those which do refer to it, including the special work on the Signs and Diseases of Pregnancy, by the late Dr. Tanner, not more than a page is devoted to this topic; and in all nearly the same explanation of the causes and the same meager hints as to treatment are found. The only exception to the above remark is the work of Dewees on Females (not his Midwifery), in which this affection is discussed pretty fully, and its treatment miutely detailed, with all the practical sagacity which characterized everything from the pen of that eminent man. I think I may say, without qualification, that since the article by Dewees was written, now nearly half a century, nothing of value, either in English or French, has been added to the literature of this subject. If the young practitioner then turns to his surgical authorities, he will find no special discussion or special direction for treatment of this affection occurring as a consequence of or a complication with pregnancy and parturition. Yet under these circumstances hemorrhoids may almost be regarded as having a distinctive pathology, while the treatment must be essentially modified by the especial condition with which it is associated. During gestation we have as a predisposing cause the pressure of the gravid uterus upon the rectum, which retards or prevents the return of the blood from the hemorrhoidal plexus of veins to the inferior mesenteric veins. But this exists as a cause in every pregnant woman, and therefore some other element seems to be necessary for the development of the disorder, and this may be either constipation or diarrhœa. In constipation there is probably the same atony of the coats of the hemorrhoidal veins as exists in the muscular coats of the rectum, and the pressure of accumulated fecal matter contributes to make these veins varicose, and, if long continued, to develop the hemorrhoidal tumors. The effect of a purgative is to stimulate an abnormal peristaltic action in directly an opposite direction to the returning blood of the hemorrhoidal veins. Some who are subject to piles are never constipated, but have habitually a loose, relaxed condition of the bowels, the same atony of the venous coats resulting from the irritation and exhaustion of diarrhœa as exists in constipation. So therefore either constipation or diarrhœa may develop the hemorrhoids. If the hemorrhoidal veins have become varicose during the later periods of gestation, the tumors may be developed by the long pressure of the fetal head on the rectum during labor. The hemorrhoidal veins sometimes swell enormously at this period, as they are probably weakened by the distension they have suffered during the progress of the labor, and they regain the power of contracting at this time with great difficulty. In many patients the hemorrhoids are first developed by the action of the purgative given two or three days after confinement.

I will now describe the treatment which I have found the most successful in each of the above conditions, and which I have substantially taught in my lectures to students for more than twenty years past.

When hemorrhoids are developed during the later periods of pregnancy, the indications are obviously to counteract the constipation or the diarrhœa, and to stimulate and to restore the tonicity of the hemorrhoidal veins. The inquiry will then naturally suggest itself, have we any agent or combination of agents in the materia medica capable of effecting these results? I know of no article which so clearly and positively produces these two results as aloes, and on this I have mainly relied. I am well aware that the general voice of the profession is against the use of aloes where there is any tendency to hemorrhoids. That "aloes is contra-indicated by hemorrhoids" is not only the doctrine of the "Dispensatory of the United States" (Wood and Bache), but it is also the opinion of most writers on materia medica from ancient times down to the present day. "Fuchsius was of opinion that of one hundred persons who should take aloes frequently as a laxative, ninety would be attacked with piles. Murray blames physicians who are induced by the gentle and certain action of this medicine to expose their patients to so serious a consequence. It was to this purgative that Fonsecha attributed the prevalence of piles among the inhabitants of Padua, and Stahl makes a similar statement in regard to the people of Hamburg. Calvin is cited as a prominent example of this mischief produced by aloes; for this celebrated reformer is said to have died ultimately from the effects of the piles which it gave rise to; but as he was of a frail constitution, subject to quartan ague, gout, and gravel, the part which aloes bore in his demise may reasonably be judged to have been small."* But these opinions have not been accepted by all; for Cullen, Sir Benjamin Brodie, Trousseau and Pidoux, and others, have doubted whether aloes is productive of piles, but attribute this infirmity not to the medicine, but to the constipation which aloes is used to remove. I will, however, parenthetically say here, from my own observation, I am convinced that aloes will, under certain conditions of the system, and in certain doses, develop piles. The special property of aloes is "to excite the muscular contractility of the colon and rectum," and "to stimulate the venous system of the abdomen, and especially of the pelvis." That these are the effects of this agent I have not only the authority of special writers on therapeutics, as Pereira, Wood and Bache, and others, but I believe the general experience of the profession also will confirm the assertion. It would seem therefore

* Stille's Therapeutics and Materia Medica, Vol. II., page 571.

that the use of aloes for the cure of hemorrhoids in pregnant women would have suggested itself from *a priori* reasoning; but I am not aware from anything that I have read that it ever has. I suppose that the general impression that aloes is contra-indicated where there is any tendency to hemorrhoids, and that it possesses emmenagogue properties, has had great influence in preventing this. In my own case the use of this article for this purpose was the result of gradually accumulating observation rather than from any reasoning on the subject.

In the early days of my professional life I was engaged to attend a woman in her confinement who suffered from obstinate constipation. I prescribed for her Dewees's pills. At the time of her confinement she mentioned that in her former pregnancies she had suffered very much from piles, but that my pills had cured them. If I had known of her hemorrhoidal tendency I should not have given these pills, and I was therefore quite surprised by her statement, as the result seemed so contrary to all that I had been taught. From this time I began to experiment as to the effect of aloes in the treatment of hemorrhoids, associated with constipation, in the pregnant; and for many years past I have constantly made use of aloes for their cure, whether the hemorrhoids were the result of constipation or of diarrhœa. I give it, combined with other agents, according to the special indications of each case, and in such doses as I learn by experience of the peculiar idiosyncrasy of the individual is necessary to secure one easy, free, daily evacuation of the rectum. Some require a grain morning and evening, while in others a half grain is sufficient. In anæmic patients I combine the aloes with the sulphate of iron. In the two last weeks of gestation I always combine it with the extract of belladonna.* The following is a frequent prescription with me:

R. Pulv. Aloes Soc., . . . }

Sapo. Cast., . . . . . . } each scru. i;

Ext. Hyoscyami, . . . . . half dr.;

Pulv. Ipecacuan., . . . . . gr. v.

M. Ft. pil. (argent.) No. 20. S. One, morning and evening.

When the patient is anæmic, I add to the above one scruple ferri sulphat. Some ten days or two weeks before the supposed time of labor I substitute the extract of belladonna, ten grains to one scruple, for the extract of hyoscyamus. When the hemorrhoids are associated with an irritable rectum, and frequent, small, teasing, thin evacuations, I substitute for the hyoscyamus a small quantity of opium, giving a smaller quantity of the aloes, as in the following formula:

* *Vide* American Medical Monthly, January, 1861, "An effort to shorten the duration and diminish the pain of the first stage of labor," by the writer of this article.

R. Pulv. Aloes Soc., . . . }
Ext. Opii Aq., . . . . } each gr. x.
Sapo. Cast., . . . . . }
M. Ft. pil. No. 20. S. One, morning and evening.

It is unnecessary for me to multiply formulæ, as the general principles by which I am guided will be sufficiently evident from the above.*

In some cases I have not been consulted, and have not known of the hemorrhoidal tendency of the patient, until my attendance during labor. I have seen the hemorrhoidal tumors sometimes become very large during the labor. Dewees says: "Much may be done during labor to prevent a severe spell of piles by the accoucheur making a firm pressure upon the verge of the anus with the palm of his hand, guarded by a diaper, during the progress of the head through the external parts, and by carefully returning them after the expulsion of the placenta, as the sphincter is now fatigued, and will not oppose their descent." I have frequently tried this expedient, but I cannot say that it has been very successful, as the tumors soon come down again, and under these circumstances they are very apt to become strangulated, inflamed, and cause a great deal of suffering. When I find this condition of things, I have within a few years past adopted the plan of forcible dilatation, recommended by my friend and colleague, Prof. Van Buren. My method is this: the patient being fully under the influence of chloroform, I select the moment after the delivery of the child and before the placenta is brought away. I push back the tumors within the sphincter, if I can readily; if not, I leave them alone, and introduce both thumbs, back to back, well in the sphincter, and opening them as wide as possible I draw

* My friend and colleague, Prof. Wm. T. Lusk, has given me a memorandum from his note-book of the Clinical Lectures of the late Prof. Oppolzer, of Vienna, in 1868, which particularly struck him, as when a student he was familiar with my teaching on the subject. He says: "At the beginning of the hour Professor Oppolzer was wont to rapidly examine and prescribe for a large number of out-patients. Many of these were Polish Jews, drawn to Vienna by the great fame Oppolzer enjoyed in the treatment of hemorrhoïds, an infirmity to which the Jews of that region, owing to sedentary habits, are specially liable. His prescriptions were: when piles are associated with constipation, aloes and quinine; without constipation, aloes and sulphate of iron. For bleeding piles:

R. Ferri Sulphat., . . . . . scru. i;
Ext. Aloes Aq., . . . . . dr. i;
Ext. Taraxaci, q. s. ft. pil. No. 60.

S. One, morning and evening, and increase to three a day if necessary."

I will also add the following sentence from Dr. Chambers's Restorative Medicine, page 58: "Take, for example, aloes. It is a purgative, evacuating effete tissue; but what a bracing effect it has upon the mucous membrane of the lower bowel, restraining its oversecretion of mucus, and restoring the elasticity of the congested blood-vessels."

them through the sphincter. During this time I have firm pressure made on the uterus by an assistant, and in several instances the operation was followed by the sudden expulsion of the placenta from the vagina. I direct the following ointment to be applied twice daily to the tumors, and well up in the rectum:

| | | |
|---|---|---|
| R. | Ung. Gallæ Co., . . . . . . | oz. i; |
| | Ext. Opii Aq., . . . . . . . | scru. i; |
| | Sol. Ferri Persulph., . . . . | dr. i. |
| M. | Ft. Ung. | |

The result has been in every instance that the tumors have gradually disappeared, and the patients have had very little suffering from the operation.

Where hemorrhoids come on after labor, the suffering is generally much greater than when it occurs during pregnancy. They are very often induced by the action of the purgative given two or three days after confinement.

It is now many years since I have been convinced that castor-oil was one of the worst agents that could be used as a laxative when there is a tendency to piles, as in many instances I have seen its action develop them. For many years I have annually spoken of this to the medical class before whom I have lectured, and I have received many letters from former students corroborating my statement by their own observation. But I have never seen this alluded to, except in one work—viz., Hardy and McClintock on Midwifery and Puerperal Diseases—who incidentally make the following remark: "We may first observe that castor-oil is ill suited for patients who have hemorrhoids, being very apt to produce in them tenesmus and considerable irritation of the rectum." I may add the following from Quain:* "Common opinion has assigned to castor-oil a character for blandness (probably because of its being an oil) to which it is not entitled. It is an efficient purgative, but, except when given in minute quantities, it usually irritates the rectum."

In Wood and Bache's Dispensatory (article *Castor-oil*), we find the following: "Some apothecaries are said to use it as a substitute for olive-oil in unguents and cerates; but the slightly irritating properties of even the mildest castor-oil render it unfit for those preparations which are intended to allay irritation." It is curious that its irritating action on the mucous membrane of the rectum has not attracted more attention.

* Diseases of the Rectum, page 16. In the case of a friend and classmate of mine, who is suffering from epithelial cancer of the rectum, and is now under the care of Prof. Gross, of Philadelphia, the first symptom of his disease followed the purgative action of castor-oil. A second dose of this medicine, a few days subsequently, produced still more intense and persistent suffering.

In those who have, or are predisposed to have, hemorrhoids, I give the following on the second day after confinement:

R. Magnesiæ Sulph., . . }

Magnes. Carb., . . . } each half oz.

Potas. Sup. Tart., . . }

Sulphur. Sublim., . . }

Mix thoroughly. S. One, two, or three teaspoonfuls of the powder before eating in the morning.

This powder produces a soft evacuation, without pain, even when the hemorrhoids are inflamed. By procuring a daily evacuation with the powder, and the use of the ointment before mentioned, I have found the hemorrhoids in puerperal women soon cease to give trouble.—*American Practitioner.*

---

*Signs of Death.* By HENRY HAACKE, Cincinnati.

Johann Ludwig Casper, in his exhaustive work on Medical Jurisprudence, after referring to Frank's statement regarding the ease with which the conjunctiva may be separated from the cornea, and to Larcher's black spot on the sclerotica, as *scientific curiosities*, says, emphatically, that *the well-known signs of death are fully sufficient for a diagnosis.* He enumerates the following stages between death and decomposition.

1. *Respiration* and *Circulation* have ceased.

2. Immediately after death the *lustre of the eyes* disappears. Who has not, he says, on raising the lids of a deceased person, noticed that peculiar, lifeless *stare*, which cannot be described? Light, of course, has no effect on the pupils.

3. A reaction cannot be brought about by any means, in any part of the body. We say nothing, here, of the experiments with electricity, because they do not properly belong to this subject.

4. The entire body grows *pale.* Still, persons that had, in life, a marked florid countenance, retain a good deal of color for many days after death. So, too, the red or livid margins of tumors do not change to the pale color of the corpse. Nor do the red, black or blue marks made by tattooing disappear. Again, if there was present, at death, an *icteric* color, it does not change to white; and, finally, all marks of blows or bruises retain the color which they had at death.

5. *The animal heat* which existed at death is retained for a time, because the cellular tissue is a poor conductor of heat. This is especially true of *fat* corpses, which, *ceteris paribus*, remain warm longer than those that are lean. But, in general, there are other circumstances that influence the retention of animal heat, such as the temperature of the place where the corpse is, and the manner in which death was brought about. It is well known how soon corpses grow cold in water, which, even in the hottest of summers, is colder than the air. In privy vaults and heaps of manure, a corpse naturally retains heat longer. The same must be said of persons that remain covered with bedding, after death. Persons who died of suffocation retain the animal heat notably longer than others. *It may be laid down as a general rule that in the great majority of corpses, the animal heat as far as the sense of feeling is concerned, disappears in from eight to twelve hours.* In experiments with the thermometer this time is, on an average, doubled.

6. Immediately at and after death there occurs, also, a general *relaxation* of the muscles, the earliest symptoms that prove the extinction of the *turgor vitalis*, and which is soon followed by other symptoms. *A corpse that shows only the signs enumerated* (1 *to* 6) *may be regarded as that of a person who has been dead for from ten to twelve hours.*

7. A valuable proof of the extinction of the *turgor vitalis* is furnished by the softening and yielding of the *eye ball.* This may be distinctly noticed, in all corpses, after a lapse of from 24 to 30 hours; sometimes sooner, whilst the living eye ball resists the pressure of the finger under all possible circumstances, even in patients attacked with cholera and in persons just dying; in from 24 to 30 hours after death this resistance has ceased. The bulb *yields*, becomes soft as butter, until at an early stage of decomposition it bursts and pours out its liquid.

8. The same cause, i. e., the extinction of the *turgor* brings gradually about the well known *flattening of the muscles* in those parts on which the corpse lies, not only, therefore, in the buttocks and calves, but also in the lateral portions of the upper and lower extremities, the cheeks, the anterior portion of the thighs, etc., according to the position in which the patient died or which he retained after death.

9. *Hypostases*, the result of the subsidence of the blood in the capillaries, according to the law of gravitation. They are, for this reason, found on the entire posterior (inferior) plane, the back, the *nates*, and the calves; but frequently, also, in the face, on the ears, on the lateral portions of the breast and the extremities. It is plain, therefore, that all hypostases may and do occur also on such unusual anterior and lateral portions of the body, as the anterior region of the stomach, etc.; and in such cases we can determine, with a great degree of certainty, the position in which the deceased must have been at or soon after death.

These hypostases become visible on the corpse in from 6 to 12 hours after death, in rare cases even before that time. They gradually increase in extent, until decomposition sets in. *They are, in themselves, a perfect sign of real death.* We must distinguish *exterior* from *interior* hypostases.

### EXTERIOR HYPOSTASES.

Hypostases of the sub-cutaneous cellular tissue. *Todtenflecke* (death spots) constitute a significant syptom of death. The unskilled are in danger of confounding them with the marks of bruises, blows and the like, from which, however, they may easily be distinguished by incisions. An incision with the scalpel, in a *Todtenfleck*, will never reveal diffused liquid or coagulated blood at the base of the incision, at most a few small points of blood caused by several cutaneous veins, whilst an incision in the very smallest mark of a blow or bruise is followed by a diffusion of blood. This being an *infallible*, and the only, diagnostic means of distinguishing exterior hypostases from the marks of blows and bruises, it ought never to be neglected.

The color of exterior hypostases differs little from that of the crawfish, of copper or a bluish-red. As may readily be understood, they are never in the least elevated above the cuticle. Their form is very uncertain,—sometimes striped, sometimes round, in other cases angular. At first they are found only here and there on the corpse, of the size of a walnut, an apple or a dinner plate, until, gradually, they become confluent and cover entire portions of the body, such as the half of, or the entire back. Age, sex, constitution, and the mode of death, have no influence on their formation; they are found even in cases of death caused by an excessive loss of blood.

### INTERIOR HYPOSTASES.

These occur more especially in the following organs:

1. In the *brain*. Here their presence is evinced by a fullness of the veins of the *pia mater*, which, in cases of a general abundance of blood in the cavity, is yet notably more marked than usual, and, in cases of anæmia, may still be easily seen. These hypostases of the brain occur even in cases of death from loss of blood. It is important not to confound them with hyperæmia of the brain (apoplexy).

2. The most constant interior hypostasis is that of the lungs. Orfila says it becomes visible in from 24 to 36 hours after death; but it occurs really much sooner, and at the time when a subsidence of the blood takes place in all the other parts of the body. The entire posterior of both lungs, about one-fourth of the complete parenchyma, is, in corpses lying on their backs, of a much deeper color than other portions, and an incision in the lungs, even in cases of anæmia, reveals a notable fullness of blood. This is so remarkable, that the unskilled are easily led into errors of diagnosis as to the cause of death, e. g., apoplexy of the lungs, pneumonia, etc. This will especially occur where the blood is very dark, and in cases of more or less œdema of the lungs.

3. Among the organs of the abdomen, hypostases occur, generally, in the *intestines*, and in the *kidneys*. They are quite common in the intestines, especially in the portions located in the pelvis. The bluish-red color of the inferior portion can deceive only the inexperienced. The difference between places reddened by hyperæmia and inflammation, is shown by the color, the absence of lustre, and by the location.

4. In the *kidneys* we find hypostases, principally at the posterior half, in cases where the corpse has remained on its back. They are, therefore, easily distinguished from a general plethora of these organs.

5. The hypostasis of the *medullary substance* of the *spinal cord*, has, up to the present time, been scarcely noticed, and yet it is important, because it, too, may lead to errors of diagnosis. It is shown in the veins of the *pia mater*, and is the more frequently mistaken for *meningitis*, as its occurrence is almost unknown to those engaged in the examination of corpses, because of the difficulty experienced in opening the spinal cord and the consequent rarity of such an operation. The novelty of the case easily misleads to the assumption of inflammation, especially when it is known that the back has been subjected to acts of violence. The truth of this assertion may be readily demonstrated by examining a corpse that has lain on its back for some days, with reference to this hypostasis.

### COAGULATION OF THE BLOOD AFTER DEATH.

6. The *heart* is not subject to hypostasis. Still, the heart, more than any other organ or vessel, presents, in the so-called polypi of the heart, a sign which is of great importance in forensic diagnosis. It is expedient, therefore, to notice them here. These polypi are, as is well known, simply *coagulated blood*. That this coagulation in the heart sometimes takes place even *before* death, we may admit in cases where the death agony is of long duration, but certain it is that in the vast majority of cases it does not occur until after death, during the gradual escape of animal heat. Bock* and Donne † even go so far as to fix the time when this coagulation commences at about four hours after death, but this can, at most, be considered only as the *average* time, for the tendency of the blood to coagulate varies according to its concrete quality. Hoppe ‡ has demonstrated that, *ceteris paribus*, the coagulation of the blood will be accelerated when it is thin and watery, (hence the rapid coagulation after loss of blood and in cases of hydræmia),

* Gerichtl, Sectionen, 4 Aufl. Leipzig, 1852, 5. 19.

† Donne, Cours de Microscopie, p. 52.

‡ Hoppe Seyler, Hdbch. d. Chem. Analyse. Berlin, 1865, p. 306.

and that it will be retarded when it is deficient in oxygen and rich in carbon. Now, since it must be admitted, that the blood does not coagulate all at once and simultaneously with death, but that it requires a certain time to go through the process of coagulation, it is clear that the doctrine *that the blood, after death, can no longer coagulate*, which is so often repeated, and was quite recently defended by Tardieu,* is not correct, and the assertion that, because the blood at the base of a wound is found to be coagulated, therefore this wound must necessarily have been inflicted *before* death, cannot be maintained.

### CADAVERIC RIGIDITY.

10. The last sign of the earliest period of death, and which always precedes the first stages of decomposition, is *cadaveric rigidity*; the well known shortening and thickening of certain muscles, especially of the flexors and adductors of the extremities, including the fingers and the lower jaw, which causes them to feel hard and firm. It begins in the neck and lower jaw, extends, subsequently, to the muscles of the face, throat, breast, and upper extremities, and finally, the lower. This rigidity disappears, in general, in the same manner, and after it has once disappeared, it does not occur again, but the corpse becomes flexible as before. Cadaveric rigidity commences, as a rule, in from 8 or 10 to 20 hours after death; in rare cases, sooner. Caspar † says that in one case he noticed cadaveric rigidity of the lower jaw in 2¼ hours after death, and of the arms in 6½ hours. It is necessary, therefore, to be cautious in determining the time of death. This rigidity may continue much longer than from one to nine days, which is the usual time. It seems to be certain, that in cases of narcotic poisoning it is either feeble or only of short duration. Tourdes,‡ who examined two persons, killed by lightning, speaks of rapid and general rigidity, in both cases. In the immature fœtus, mature *neonati* and small children, it is comparatively feeble and of short duration. Caspar denies the statement of Sommer that this is also the case in the aged. He considers it an error to maintain, as is often done, that in cases of death from suffocation, this rigidity does not occur at all, or at a later period, or only for a short time; they do not differ from ordinary cases. That a low temperature and alcohol favor a longer duration of rigidity cannot be doubted. Caspar noticed it in several cases of death during intoxication as late as the fourth, seventh, eighth, and even ninth day.

* Tardieu, Etude medico-legale sur l'infanticide. Paris, 1868, p. 71.

† Caspar, Hdbch. d. gerichtl. med. Berlin, 1871, p. 27.

‡ Tourdes, relation medicale de l'accident occasionne par le foidre. Strasbourg, 1869.

Where the *rigor mortis* is of longer duration, it is not uncommon to notice, in connection with it, discoloration caused by decomposition. An advanced state of decomposition alone does not, therefore, put an end to it. That it is in no case ever *entirely* absent, seems to be well established. The *rigor mortis* cannot well be confounded with the rigidity caused by freezing. A frozen corpse is as stiff as a board, from head to foot, whilst in *rigor mortis* the extremities are, especially in the joints of the elbow and knee, still susceptible of some degree of flexion.

*A corpse that presents only the signs before enumerated* (1 *to* 10) *may be taken to be that of a person who has been dead at most two or three days.**

In order to accurately determine the *time* of death, it is of course necessary to have a correct knowledge of the various stages of the process of decomposition, but, the present article being already of sufficient length, and having, moreover, given an account of all the signs necessary to determine *death itself*, we will defer the rest of the subject to a future time.—*The Clinic.*

---

*About the Action of Derivatives.* By Dr. Med. W. Zuelzer. Translated for the Review from the *Deutsche Clinic*, by J. Henry Carstens, M.D.

The question if the so-called derivatives (epispastica and such remedies) really produce any marked effect, and what this effect is, has only lately received sufficient notice. While some deny that a marked influence is produced, Sabatier has noticed a beneficial effect in a large number of diseases from the external application of derivatives. To say little of the few observations made by Whytt, who noticed that vesicants not only do not increase the number of pulse beats, but that they actually lessen it, Naumann recently has examined these actions more thoroughly in a brilliant work, "Ueber die Wirkung der Epispastica." Naumann found that the therapeutic effect of these agents was produced principally by reflex action, therefore, by means of the central organs of the nervous system; that active irritation of the skin lessened the action of the heart and blood-vessels; weak irritation, however, increased the same. That the point of application was nearly always of no importance, and that the hyperæmia accompanying the irritation of the derma had of itself no bearing on the action of irritants. However reliable these conclusions may have been proven experimentally, they seem insufficient to account for the real derivative action noticed in private practice of such perma-

* Caspar, p. 30.

nent vesicants as cantharides, setons, etc. As even Sabatier says: "Experience proves that it is not sufficient to apply, without consideration, a counter-irritant to the skin, with the intention of drawing the irritation from the affected internal organs, but that at this or that spot of the skin the derivative agent must be applied."

The changes produced by vesicants and other irritants have not been demonstrated up to the present time. In the following paragraphs I shall describe some trials made with this object in view.

On one side of the back of a strong rabbit cantharidal collodium was applied repeatedly for two weeks, over a spot three and a half by two inches in size. On dissection the skin superficially appeared partly ulcerated and partly scabbed over, the under part of the skin's blood-vessels were dilated and filled. The fat had entirely disappeared, while the healthy side showed a thick layer. The superficial muscles were hyperæmic, with extravasations, and, therefore, naturally kept their electric re-active power longer than those on the other side. The deeper muscles were, however, very pale, if compared with those on the healthy side; also the thorax wall, on the inner side of which the difference could even be shown. This remarkable lack of blood even extended to the muscles of the thigh.

On repeated trials of this experiment even the lung was found anæmic if compared with the healthy side.

A seton was then applied to the fore part of the knee of a young rabbit, and left there for four weeks. Ulceration set in quite profusely; locomotion was only partially interfered with the latter part of the time. At the section the sinus caused by the seton was filled with pus, and the surrounding tissue inflamed and infiltrated with purulent matter. A pretty large cavity, filled with thick, cheesy pus, opened upwards. All muscles in the neighborhood of the joint were perfectly anæmic, and all the component parts of the joint (ligaments, capsules, etc.,) showed marked lack of blood, while these parts on the sound leg showed the rose red color. Graver changes of structure in the cartilaginous tissues I could not prove; nor could I, after repeated trials, find any influence on the growth of the bones; these, however, showed no difference in size if compared with those of the healthy side.

Finally, on the fore-arm of a young strong man cantharidal plasters an inch square were applied successively; when the wound caused by one was healed, a fresh plaster was applied. In a short time the arm began to swell, and showed a marked increase of temperature. Later, a light lymphangitis was developed, which lessened the usefulness of the limb still more.

By these few experiments it is shown that epispastics, if used for some time, cause a marked derivative action, and that this action has a bearing on the amount of blood. This proves the theory of the action of derivative remedies adopted by the profession to be correct.—*Detroit Review.*

*The Medical School of Berlin compared with that of Vienna.* By D. F. Lincoln, M.D., Boston.

To the crowds of young Americans who study, or look forward to studying medicine in Germany, the central point of attraction is undoubtedly the "Allgemeines Krankenhaus" of Vienna. Americans do not often deceive themselves when there is a question as to the practical advantages to be gained, and, upon the whole, Vienna must be admitted to deserve the popularity it enjoys. But there is another aspect of the question, of course. It would afford an exceedingly instructive comparison if Vienna were placed upon one side, and upon the other were grouped, by way of contrast, the leading medical schools of Germany—Berlin, Leipzig, Munich, Wurzburg, Breslau, Heidelberg, and others. What the points of contrast would be, I am unable to do more than hint; since the object of the present letter is mainly a description of the School of Berlin.

It is said, with apparent truth, that the physicians of Vienna differ from those of Berlin in devoting themselves much earlier and much more completely to specialties. Hence the charge sometimes made against them, that their development is less broad and general than it should be. We know that Skoda and Oppolzer did not achieve their greatness at the expense of breadth, for both had charge of general clinics, and both were men of truly sagacious minds, free from the objectionable influence which specialism sometimes exerts upon small men. But let these two men, with Rokitansky, stand for what the Vienna School has been during the last twenty years, and let Virchow, von Graefe and Griesinger stand for Berlin; are we not struck with the fact that the latter trio is composed of *great* men, in a sense in which the expression does not apply to their Viennese colleagues?

At the present time, in Vienna, Stricker is a man of purely special research. Billroth is perhaps the broadest, probably the most "practical" man in the School; he, however, is not an Austrian, but was summoned from a German university. Hebra and his followers, Kuhn, Neumann, Sigmund and Zeissl; Meynert; Arlt and Jaeger; Politzer and Gruber; Widerhofer; Benedikt and Fieber; Wedl, Schrotter, and others that might be named, form a brilliant group of men who are almost entirely devoted to specialties. The anatomists, pathologists, chemists and physiologists of Vienna are of equally deserved fame. But, outside of this remarkable array of talent, we find but one or two names of eminence in general medicine; a fact which strikingly coincides with the extraordinary poverty of opportunity for general clinical study, of which the students make earnest and just complaint. Inferences apart, Vienna is the school for specialties.

As a general rule, the value of teaching depends on the character of the teacher much more than on the amount of material at his disposal. There is enough material in Vienna, if judiciously used, to serve for thrice the actual number of students. It is used, or it is wasted, as the case may be; and if wasted, it harms the quality of the teaching more than mere poverty possibly could. If a Virchow had at his disposal fifteen autopsies a day, instead of five, no doubt he would know what to do with them; but upon young men the effect of such an overplus of matter is apt to be seen in superficial and careless habits. As a case in point, let me quote the remarks of a correspondent who has studied ophthalmic surgery in Vienna, and who now writes me from Breslau to the effect that the instruction in his specialty is most excellent: "the material is not very abundant, but what comes is most thoroughly worked up, which I consider much better than the mass of half-digested (?) material one gets at Vienna."

There is a good deal of justice in the general impression among us at home, which makes Virchow the representative of the Berlin school. Since the death of Graefe, Virchow stands undoubtedly head and shoulders above his colleagues in that city. Add to this the fact that he possesses an admirable gift of teaching, that he is master of an excellent laboratory, and abundant material, and is served by most efficient assistants, and it will not exceed the truth to say that for the study of pathology Berlin is incomparably superior to Vienna. If it be a prominent object with a student to make himself master of pathology, he should by all means visit Berlin before Vienna.

Virchow's lectures are about two hours and a half long; I have no doubt he could lecture six hours equally well, for he never shows a sign of flagging. On Mondays, he takes scalpel in hand and performs an autopsy before the class, making the body before him the text of his discourse; on Wednesdays and Saturdays he describes the specimens which have accumulated from the previous days. His statements are clear and simple, his voice distinct, his manner easy; he is never in a hurry, and never above the comprehension of his hearers. He enjoys a dry joke amazingly; and as he cracks it, with characteristic deliberation, he is apt to bring down the hammer upon the unlucky fingers of some of his hearers. As a teacher he leaves nothing to be desired; he was born to the office.

On Tuesday, Thursday and Friday a systematic course of microscopic pathology, lasting the whole term, is given by one assistant, under the general oversight of the Professor, and normal histology is taught in the afternoon of four days by the other assistant, the course lasting six weeks. Both of these microscopic courses are admirable, and both are fully appreciated by the British and American students. Good instruments are furnished for those of the class who do not own them.

With three hours' work in the forenoon of every day, and two hours in the afternoon of four days in the week, and collateral reading, a student need not blame himself if he finds his time wholly occupied by Virchow's courses, especially if he attends the systematic theoretical lectures which are given by Virchow on four days in the week.

The *Charite* at which most of the clinical instruction is given, is a large, massive, not unattractive building, of four stories, enclosing several ample courts planted with grass and trees. Internally its arrangements are simple, and hardly up to the modern standard, the fault of which lies in the fact of its being an old building. It is as clean, tidy and cheerful as the "Massachusetts General." The number of beds is in the neighborhood of eighteen hundred. The quadrangle known as the New Charite contains about three hundred beds, half of which are devoted to insane patients, and the remainder are occupied by the syphilitic and by prisoners and convicts sent thither by the authorities for treatment.

The Clinic for Mental Disease deserves especial mention. Griesinger introduced the "non-restraint" system in the wards under his charge, and it was through his efforts that the government was induced to permit the establishment of one of the first public clinics of the kind in Europe. His successor, Westphal, is a man in the prime of life, of quiet and gentlemanly manners, and a master of that tact which is indispensable in the care of the insane. His speech and intellect are quick and vivacious. As a lecturer, he is very systematic, and economizes, as far as possible, the lecture-time. Without placing him among the great thinkers of the day, I must still rank him among the best of lecturers upon medical subjects. To be sure, the lecture can never be dull; for one or two patients are always brought in and examined in the presence of the class. One must see this done in order to understand how entirely consistent are humor and humanity. Younger students do not attend these demonstrations; in fact, a large number of the hearers are physicians, forming an audience such as one seldom sees equaled for intelligence. They are divided into sections of a dozen or so, which make the morning visit twice a week in company with the professor. I could wish that every physician and every thinking man might come in contact with the insane element of our population in the free and natural way in which it is here effected. The wards resemble those of an ordinary hospital, and the degree of restraint exercised is no greater than that required in a children's ward at home. The patients have the liberty of two or three rooms, opening freely into a long, lighted hall or corridor, which is locked at both ends. These locks constitute the only restraint that the patients experience. It would take too long to describe the "non-restraint system." But let it be remarked, in passing, that during the four years of Prof. Westphal's superintendence, the strait-jacket has never been applied to a single one of the hundred and fifty insane, of all classes and conditions, who fill the wards of the Neue Charite; and that the result of this radical reform has been a wonderful increase in the quietness of the wards. Furious patients can be pacified, in almost all cases; and in an extremity, the cell, or chloroform, or chloral, remain as the most potent known soothers of maniacal frenzy. No patient is kept longer than 24 hours in the cell.

Two lectures a week are given on Mental Disease, and one upon Nervous Disease. The wards for patients of the latter class may contain fifty or sixty beds; the cases are extremely instructive, and are exhibited to the class in the same way as the insane cases.

Worthy to stand in the first rank, and to form an object of leading consideration with the student who is looking towards Germany, is Traube, the acute and indefatigable teacher of clinical medicine, less popular than Frerichs, his superior in rank, but possessing altogether a finer and stronger mind than the latter. It is not necessary to describe him at length, but only to say that his name deserves mention near the name of Virchow, in enumerating the attractions of Berlin.

Anatomy may be studied to advantage. The building devoted to this purpose is large, handsome and commodious. The dissecting rooms contain about forty tables, some for whole subjects, some for portions; they are well lighted and ventilated, and the supply of material is abundant. From these rooms a trap opens up into the lecture hall, which contains about three hundred seats, and is a model of good arrangement. "The parts are given out every Monday. I was there this morning. There were twenty legs with half of the pelvis, for the crural nerves, fifteen or more arms, with half the thorax for the brachial plexus, a dozen half-heads cut perpendicularly for the brains, etc., six children, as many urinary organs, the bladders blown up, ready for distribution, besides other bodies and parts of bodies." Parts are distributed by lot. "Prof. Reichert passes two hours every morning in the dissecting room, going to half a dozen tables and dissecting and explaining to those around him. He lectures every day between 1 and 2. He has also his private rooms, where one or two anatomists work. Besides his course on general anatomy, he gives one on histology. His assistant, Prof. Hartman, lectures on osteology and syndesmology. The whole arrangement deserves much praise."*

Chemistry is very well taught by Hofmann and others. The laboratory is in the Georgen Strasse.

* From a note from Dr. Coolidge, to whom and to Dr. Putnam I desire to express my thanks.

Materia Medica is also well taught by Liebreich, the discoverer of the virtues of chloral.

In surgery, we find Langenbeck, Bardeleben and others; but there are several large cities where they speak English, in which surgery is better taught than in Berlin or Vienna.

Skin diseases and syphilis are taught here clinically, but there are other places for their study far better.

The lying-in department of the Charite contains two hundred beds. The subject is not especially well taught.

Tobold has a class in laryngoscopy, which is decidedly poor.

The eye is taught by Schweigger, an able man, and Hirschberg. The ear by Lucae.

In electro-therapeutics, the names of Eulenberg and Hitzig are well known, and their lectures (private) are better worth attending than those in Vienna.

In physiology, the names of Du Bois-Reymond, Rosenthal and Munk, require no comment, except to say that they are clear and excellent lecturers.

In all, one hundred and seventeen courses, great and small, are advertised upon medical subjects, not including zoology, botany, mineralogy and chemistry.

Last year there were 2,113 matriculated students in Berlin, of whom 454 were studying medicine; 456 were foreigners. Besides these, 519 were in the field with the army. The medical students of Berlin are certainly much more prepossessing, both in appearance and manners, than those of Vienna. Indeed, they are as well-looking and gentlemanly a body of men as is often seen.

It is proper to add, that the cost of living in Berlin, though prices have risen since the war, is still much less than in New York. As an instance, a good furnished room costs from eight to twelve thalers a month.

The winter term commences about November 1st, (nominally in the middle of October), and lasts till Easter. The summer term lasts till the middle or end of July. Few persons would willingly remain during the month of August.—*Boston Medical Journal.*

---

*The Possibility of the Diagnosis of Syphilis by means of the Microscopic Examination of the Blood.* By Dr. LOSTORFER, of Vienna. Translated from the Wiener Med. Presse by James R. Chadwick, M.D.

The general interest which has greeted the matter induces me to give here an abridgment of an article destined for publication elsewhere.

In the examination of the blood from syphilitic patients, I employed the following method. A drop of blood of a syphilitic upon a slide covered with covering-glass, was placed in a moist chamber for preservation, and was daily searched with a Hartnack ocular 3, objective 10 a immersion.

The result of these examinations, which I have prosecuted without interruption upon numerous specimens of blood from individuals presenting the most diversified forms of syphilitic symptoms, and which proved to be constant in almost every case, was as follows:

On the third, fourth or fifth day, and exceptionally after twenty-four hours, small, shining corpuscles were observed between the blood corpuscles, a part in a condition of rest, and a part in oscillatory motion. In the following days, these bodies increased in size, so that between the fourth and sixth days they had attained the dimensions of the red blood corpuscles. Most of them were ball-shaped, others irregular, many had one sprout, others several; of these sprouts a part were connected with the corpuscle by a pedicle, and a part not. On certain sprouts one or more secondary sprouts were noticed. After the sixth or eighth day, a "vacuole" formed in the corpuscles which increased in size until it was enclosed in a thin layer, as shown by its double contour. On the addition of distilled water after the vacuoles had formed, consequently after the sixth day, an increase of the vacuoles in the corpuscles, a formation of the same in the sprouts, as, also, in several cases, of long excrescences, similar to the tubular buds (Keimschlauchen) of fungi, took place. No further development have I observed, even after six weeks' examination.

The number of these corpuscles varied from 1—50 in the field of vision. At the same time, as well as for months previous to the examination of the blood of syphilitics, I searched specimens of blood from healthy persons in the same way, and in no case found the described corpuscles. Equally without result was the examination of blood from individuals suffering from typhoid fever, lupus, gonorrhœa, diphtheritic ulcers, one with eczema capitis, and one with leprosy. The appearance of these corpuscles, therefore, seems to be peculiar to syphilitic blood, wherefore I have given them the name of syphilis corpuscles. That this peculiarity of syphilitic blood provides us with the possibility of a diagnosis of syphilis I have proved, thanks to the voluntary assistance of Professors Stricker and Hebra. These two gentlemen laid before me—the former five times and the latter twice—a number of specimens of blood, which had been taken partly from healthy, and partly from syphilitic individuals, and numbered; between the third and sixth days, I brought to their knowledge the result of my examinations, with the exception of one experimental series, where the result was negative, and of a specimen here and there, which was designated as useless and set aside. In perfect correspondence with the record, I indicated the blood of syphilitic patients as syphilitic, and that of healthy persons as healthy blood.

Considering that, at these examinations, more than fifty specimens of blood came under observation, and that from these I selected about twenty as diseased, full credence can hardly be refused to this result, which I make known in accord with Professors Stricker and Hebra.

From the clinical histories of the cases examined with positive results, it is evident that the presence of the syphilis corpuscles is not connected with any particular form of syphilis, but is found in the most varied forms. As extreme points I can mention the induration when three or four weeks old, before the appearance of an exanthema, on the one hand, the node in company with cutaneous ulcers, where disease had lasted six years on the other.

As to the source whence the syphilis corpuscles proceed, and the role which is allotted them, the present examinations have given no clew. The solution of these and many dependent problems must be deferred to future examinations.—*Boston Med. Journal.*

---

*On Purulent Infection.* By M. ALPHONSE GUERIN.

The following conclusions were given at the end of a long address before the Academy of Medicine, Paris:

1. The affection which we designate as purulent infection or pyæmia, ought to be called surgical typhus.

2. Like all the other forms of typhus with which it has the greatest analogy, surgical typhus is the result of blood-poisoning.

3. This poisoning proceeds from the absorption of deleterious miasms engendered at the surface of wounds.

4. It gives rise to the formation of metastatic abscesses, and produces a lesion which has been described under the name of infarctions.

5. These infarctions, like abscesses, proceed from the action of the poison on the tissues where they are developed.

6. Although by experiments in which the circulation is obstructed by injections into the veins, one may produce abscesses and infarctions, still it is not right to maintain that these lesions cannot result from the influence of miasmatic emanations acting during life upon those parts of the body in which they are afterwards found.

7. Surgical typhus is an essentially different disease from putrid infection.

8. These two affections, although different, belong to the class of septicæmia.

9. Traumatic fever ought not to be ranked in the same class. There is nothing to show that this affection results, as has been maintained, from the absorption of a poison.

10. Surgical typhus is an infectious disease; that is, it is contagious by the air.

11. The poisoning agent cannot yet be designated except by the vague term of miasm What has been described under the name of sulphate of sepsin seems to be nothing more than a material acting like all putrid stubstances.

12. Sulphate of sepsine has been found in yeast.

13. In order to prevent or hinder the production of surgical typhus, one ought, when the patients cannot be isolated, to guard the wounds from the contact of contaminated air. Dressing with cotton wool seems to be the most certain means of attaining this end.

14. When surgical typhus exists, the best medicinal agent is sulphate of quinine, given in doses varying from two to four grammes.—*Gazette Hebdomadaire.*—*Eclectic Journal.*

---

*Treatment of Fracture of the Clavicle.* By LEWIS A. SAYRE, M.D.

Dr. Lewis A. Sayre, of New York, has finally reduced the treatment of this fracture to two strips of adhesive plaster, without any axillary pad; and as such he now gives it to the profession as the simplest and most efficacious yet devised.

His method of keeping the inner portion of the clavicle from riding over the outer portion, is by putting the clavicular portion of the pectoralis major muscle on the stretch, and compelling it to pull the clavicle in place, and thus overcome the tendency of the clavicular portion of the sterno-cleido-mastoid to elevate it, which it will always do unless this precaution is taken. After drawing the arm backward and retaining it there by a strip of adhesive plaster, pass another piece of plaster from the well shoulder across the back, and by pressing the elbow well forward and inward, the first plaster round the middle of the arm is made to act as a fulcrum, and the shoulder is necessarily carried upward, outward and backward, and the plaster being carried over the elbow and forearm (which is flexed across the chest) to the opposite shoulder, the place of starting, and then secured by pins or stitches, permanently retains the parts in position.

Dr. Sayre formerly commenced the first plaster on the inner side of the biceps; but he found that that muscle would roll around and the plaster would lose its hold, requiring to be renewed occasionally; and if it completely encircled the arm for the purpose of a stronger attachment, it would arrest the circulation, and thus prove dangerous. He uses strong and good adhesive plaster

(Maw's moleskin is the best) cut into two strips, three to four inches wide (narrower for children.) By this plan of treatment the patient is only detained from his daily avocation a sufficient length of time to properly adjust the strips of adhesive plaster.

In one instance, a prominent lawyer of New York City slipped upon the ice and fractured his clavicle, on the way down town. He was brought to his office. Dr. Sayre dressed him in the manner described, at 9 A. M., and before 11 he was pleading his case in the open court. A blacksmith was brought to his office with a fracture of the left clavicle. He dressed it, and in less than an hour the patient was again working at the forge with his other arm, and continued his labor without any interruption. In both cases the union was perfect and without any deformity. In closing, Dr. Sayre could multiply these cases by many similar ones, and he therefore feels quite confident that if any surgeon will follow the plan suggested, he will have equally good results.—*American Practitioner.*

---

*The Rational Treatment of Blennorrhagia.* By J. C. VAN WYCK, M.D., of Oakland, California.

So much has been written upon the history, etiology and treatment of blennorrhagia, that little, if anything, new or instructive remains to be said, except what relates to the treatment of this, unfortunately too common, disease. Among all the ills to which flesh is heir to, I am sure there is not one which has been the subject of so few mutations in treatment within twenty years past, or has benefited less by the adoption of rational, rather than empirical procedure. That blennorrhagia—as the term is generally accepted—unconnected with urethral chancre, is purely a *local* affection no one will, I presume, for a moment deny. What, then, should be the course adopted for its treatment and cure? Experience has taught me that topical applications alone are by far the most certain and speedy; and I take issue with those who recommend a constitutional course, believing it altogether unnecessary and useless so far as the urethral inflammation is concerned. Authors differ widely as to the character of topical applications, the utility of which is acknowledged almost universally. The abortive treatment, when first suggested and tried, had many warm partisans, and still is practiced and relied upon by some practitioners. Experience has proved that strong injections of Argent. Nit. are not only useless, but in many instances positively injurious, and the profession now use it, as well as preparations of copper or zinc ordered in the form of injections, in comparatively small quantities. Experience has long since taught me that in the large proportion of cases which come under the notice of medical men, the period has passed when the abortive treatment would be available, and the resort to it proves not only valueless but absolutely pernicious. Yet when a case presents in the early part of the first stage, it is by far the best mode of treatment known *if* followed by the use of proper astringent and anodyne injections. Copaiba and cubebs in some form or other have always held the highest rank in the scale of remedial agents in this disease, and no one will for a moment presume to deny their beneficial effects. I surely do not, yet I am positive in asserting that either or both can be dispensed with rather to the benefit than detriment of the case. I see no reason why a patient should be made to swallow nauseous drugs which tend to oppress and derange the stomach, when an equally good—yes, better—end can be attained by topical applications which do not have to pass through nature's laboratory before becoming beneficial. The use of injections is regarded by many practitioners as positively injurious, attributing to them the orchitis, which is not an infrequent result of the disease; whereas had the injections not been supplanted by constitutional or other ineffective modes of treatment, the disease would have been either cured or so checked that orchitis, a natural sequence of the specific inflammation, would not occur. Somewhat more than a year since, dissatisfied with the result of all the various means previously used, I determined to make use of a remedy of comparatively recent date which had been presented to the profession as a valuable agent in the treatment of numerous affections, and more particularly those affecting the mucous membranes, viz., carbolic acid, believing, or at least hoping, that from its highly antiseptic properties it would, inconnection with some mild astringent, convert the irritating discharge into a comparatively harmless one, and at the same time act as a sedative to the inflamed surface.

The first case which presented had been fully developed some ten days; (too long a period, in my judgment, for the abortive plan to be other than useless, if not deleterious.) I ordered the following to be used as an injection to the amount of half an ounce three times a day:

| | | |
|---|---|---|
| R. | Ac. Tannic, | scru. j; |
| | Ac. Carbolic, | scru. ij; |
| | Glycerinæ, | oz. j; |
| | Aquæ pur., | oz. vij. M. |

This patient was compelled, from the nature of his avocation, to be constantly on his feet, exposed to cold and wet, he being a carriage washer. A few applications served to remove entirely the severe burning sensation on urinating, and change very decidedly the character and quantity of the discharge. Before finishing the second bottle he declared himself perfectly cured, and had no return of an unpleasant symptom or sensation. From the first day he improved so rapidly and satisfactorily that I did not deem it necessary to adopt any other measures for his relief.

Since that time I have treated eight cases in all of blennorrhagia, and my partner, Dr. Cushing, has had three patients suffering with this affection under his care. Two of those treated by me were seen within twelve hours after the discharge was noticed. In each of these I adopted the abortive treatment, using a small piece of sponge attached to a wire and passing through a silver tube, which I introduced some two inches within the urethra and thrust out the sponge previously saturated with a solution of Argent. Nit. grs. x, to the ounce of water, with which, by a rotatory movement, I freely swabbed out the canal at least two-thirds of its extent. I then ordered the following to be used as an injection after the expiration of a few hours:

| | | |
|---|---|---|
| R. | Zinci Sulph., | scru. j; |
| | Ac. Carbolic, | gtt. xx; |
| | Morph. Sulph., | grs. iij; |
| | Glycerin. | oz. j; |
| | Aq. pur., | oz. vij. M. |

Use as an injection three or four times daily.

These eleven cases, treated in this manner, are convincing proof to me that carbolic acid, in combination with astringents and anodynes, is as near being a specific in uncomplicated cases of blennorrhagia, as any remedy believed to be such in the domain of the materia medica; and so firmly am I convinced that to it alone is to be attributed the very gratifying results which have marked every case in which it was employed, that with the next patient who comes under my care I shall use the remedy simply diluted with distilled water.

This remedy may be nothing new to the profession at large; but I have yet to see any published account of its use, and am not aware of its being used except by those to whom I mentioned the results of a treatment I adopted as an experiment, which has succeeded beyond my most sanguine expectations.—*Western Lancet.*

---

*Extracts from an Unpublished Essay on the Therapeutic Uses and Abuses of Quinine and its Salts.* By O. F. MANSON, M.D., Professor of Physiology and Pathology in the Medical College of Virginia. "Ce que j'ai vu, je le raconte."—*Maillot.*

*In Erysipelas.* Cinchona was employed by Fordyes and many others in erysipelas, and soon after the discovery of the sulphate of quinine, Elliotson made trials of it in the treatment of the same disease, in combination with wine and porter in the last stage. But it is not in regard to its employment in that period of the disease to which we wish to direct attention. It is of its use as a sedative and anti-phlogistic—as a remedy possessing the power of arresting its course, that we wish more particularly to treat. In our first experience in the treatment of erysipelas, we adopted the usual method recommended by authors, and on the introduction of the tincture of the muriate of iron, that valuable medicine was added to our remediate means, and employed with gratifying success. But there was a class of cases which this treatment did not arrest, and often signally failed in moderating in the slightest degree. These cases were marked by a very frequent, full pulse, great heat and dryness of the surface, headache, and sometimes delirium. The nervous disturbance was very conspicuously marked, and the march of the disease towards a fatal termination exceedingly rapid. Guided by a knowledge of the effects of quinine in health and disease, when freely employed, we resolved to test its powers in the first grave case presented to us. An opportunity was soon offered, and the effects of the remedy were prompt, potent, and decided. Under its action the pulse was rapidly diminished in frequency, force, and volume; the disturbed and excited innervation was calmed, and the febrile heat quickly removed.

During the late civil conflict, we employed the remedy in idiopathic, and still much more frequently in traumatic erysipelas, with invariable success. Employed early in the disease, in large doses, the quantity always increased according to the degree of fever present, the disease promptly yielded in every case. In those cases in which the disease had spent its violence, and terminated in extensive effusion and suppuration, of course we could not expect, nor did we meet with, such decided and prompt results; but even in these cases the remedy was found efficacious in arresting the further ravages of the malady, and exerted the most salutary influence in promoting recovery. In cases requiring surgical operation, I did not hesitate to cut through erysipelatous tissues, and found but little difficulty in checking the disease afterwards. The number of cases thus treated cannot be accurately stated, but there were three or four hundred at the least calculation.

The treatment adopted was simply to unload the bowels, if necessary, and then to administer the sulphate of quinine in doses of 5 to 20 grains, according to the intensity of the febrile action. On an average, 30 grains were administered in the first twelve hours; the medicine was then discontinued for twenty-four hours, and resumed if the disease continued its progress. The tincture of iron was also generally employed at the same time, and doubtless contributed greatly towards the cure. With these two remedies in the hands of the surgeon, and freely and boldly administered, I believe that erysipelas is almost as completely under control as intermittent fever.

*In Scarlatina*, Morton* employed Peruvian bark in scarlet fever. In his admirable work, among others, he relates the case of his own child, "*Filia mea charissima*, MARCIA," in which he employed it successfully. This disposes of all claims in regard to priority in its therapeutic use in this disease, but by a strange coincidence, before I had ever seen a copy of that old and neglected work, I used the sulphate of quinine in the case of my own child. Trusting that a brief recital of the case may prove useful, I will relate it as accurately as I can remember it. The patient was my own son, nine years of age. He was suddenly attacked with chilliness, followed by ardent fever, inflamed fauces, and well developed eruption on the second day. The surface was of high febrile heat, except the lower extremities; these were rather below the natural temperature. The head was hot, the face flushed, and in the afternoon he became delirious and extremely restless. His bowels had been gently opened by an aperient, and at night leeches were applied to his temples, but were followed by only slight relief; these were succeeded by cold applications to the head, mustard pediluvia, etc. He continued to grow worse, and on the next night his pulse became extremely rapid, and his extremities and cheeks became decidedly cool, the delirium continuing. Knowing the property of quinine, of diffusing the animal heat, besides its sedative and calming effects, I resolved to employ it. Ten grains were administered at a dose at night. Under its operation the child fell asleep, the pulse was greatly diminished in frequency, the extremities became warm, the febrile heat of the head and body was reduced to a normal temperature, and under its subsequent use in small doses, convalescence rapidly ensued.

I regard this case as a happy and appropriate illustration of the most valuable properties of the sulphate of quinine. Such cases are more instructive than all the experiments ever performed on dogs by M. Briquet and M. Melier, or on rabbits by M. Giacomini. We here observe the effect of a remedy in which M. Briquet, enthusiastic as he is in regard to its valuable properties, yet thinks has a tendency to produce congestion of the vessels of the pia mater. Yet in this case, in which there was as convincing proof, from the symptoms, that irritation and congestion of the brain existed, as if it had been demonstrated by an autopsy, all the phenomena of cerebral congestion were perfectly and rapidly dissipated under its influence. It remains to those, who contend that quinine excites the brain and produces congestion of its vessels, to explain its therapeutic results according to their hypothesis; we give the facts. As has been forcibly said by M. Broussais, "Le vrai medecin est celui qui guerit; l'observation qui n'apprend point a guerir n'est pas celle d'un medecin; c'est celle d'un naturaliste, ou si

* Opera Medica, I. De Febre Scarlatina, 28–35.

vous aimez mieux, d'un physiologiste etranger au but que se propose le medecin."

Dr. Edwin A. Morrison,* of Brunswick county, Va., in an interesting article "On the use of Quinine in Scarlet Fever," "offers to his professional brethren the benefits of his experience, sincerely trusting that those of them who may be disposed to try the prescription will find it as efficacious as it has proved to be under his direction."

This gentleman, after briefly relating some of his cases, gives the following as his mode of treatment: "When the first symptoms of the disease appear I order quinine, regulating the dose by the age of the patient. This is administered every two or three hours, until the system is brought decidedly under its influence. During this time I occasionally give a few grains of blue mass to move the bowels gently, and mop the throat with a strong solution of nitrate of silver. When the patient is old enough, I direct a gargle of red pepper tea and common salt to be used. I order the throat of a child to be washed with the same." The Doctor concludes by saying, "I am aware that Peruvian bark has long ago been employed in cynanche maligna, and that some writers have maintained that this is identical with scarlet fever. Hence, it may be said that there is nothing new in the use of quinine for scarlet fever. My object in this communication is not to claim originality, but to place before my medical brethren the results of my experience. I will add that my friend Dr. R. Sims, of this county, has used quinine in some few cases of scarlet fever with the happiest effect."

Dr. Morrison is one of the most successful practitioners in Southern Virginia; has been engaged in a large practice for many years, and is a gentleman whose statements are entitled to the most implicit confidence.

*In Pneumonia.* In that form of the disease assuming a periodical character, of an intermittent, but usually of a remittent type, the value of the sulphate of quinine is generally conceded. Whatever the pathology of the disease may be; whether, according to the view of some writers, it is one of the numerous local congestions or inflammations occurring in the course of periodical fever, and originating from the same cause, or merely an accidental complication of the pyrexial malady, the powers of the sulphate of quinine in accomplishing its cure are incontestable. An experience of twenty-one years in a region where this form of disease was annually present, enables me to add my humble but positive testimony to its capacity, for not only arresting the febrile phenomena, but also the parenchymatous inflammation. It is well known to

* Virginia Medical Journal, XI, 89.

the student of malarious diseases, that Morton,* J. P. Frank,† Sarcone,‡ Laennec,§ and others, had observed the wonderful powers of bark in the treatment of this disease, and that the sulphate of quinine has been found equally potent by numerous writers, but it seems to have been generally employed in reference to its supposed anti-periodic virtues; yet, since its sedative and anti-phlogistic action has been clearly ascertained, its effects on the inflammation have been found much more salutary. Constant‖ states that under the influence of large doses of quinine the hepatization rapidly disappears, and the testimony of Morehead¶ is no less decisive. This author states that "I am not acquainted with anything more striking and satisfactory in the whole range of rational therapeutics than the progressive but speedy restoration of a hepatized lung co-existing with fever of remittent type, when the exacerbations have been controlled by the adequate use of quinine." Not only in this, but in what he considers to be idiopathic pneumonia, he gave "quinine in combination with antimony in the same manner as in the febrile cases, and very frequently with the same good effect." It is true that this author was not aware of the sedative action of quinine, and gave it as an anti-periodic, yet his therapeutic facts remain the same. In Italy, we are told by M. Briquet,** that numerous cases of pneumonia are reported as having been treated successfully by Rasori, Tommasini, and their followers, and in France by M. Favier, M. Guerard, and others.

M. Grisolle,†† in his valuable work, cites a long list of authors who have used the sulphate of quinine in periodical pneumonia, and Drake‡‡ has recorded an equally high estimate of its virtues in the same malady. It is impossible to reconcile these proofs of the anti-phlogistic and sedative effects of the sulphate of quinine with the hypothesis of its excitant and stimulant action, and whether the anatomical character of these pneumonias are congestive or inflammatory, or both, they totally disprove the conclusions of M. Melier that the salt has any tendency to produce congestion of the parenchyma of the lungs.

To those who hold that periodical pneumonia is a hybrid disease, composed of inflammation of the lungs, and remittent or intermittent fever, clinical experience affords such unanswerable proofs

* Op. cit. Hist. xxi. Lugduni, 1737.

† Traite de Medicine Pratique 1, 176.

‡ Maladies observees a Naples Tome 1, 176.

§ Traite de l'Auscultation mediate 3m. Ed.

‖ Bulletin de Therap.; xliii, 481.

¶ Clinical Researches on Disease in India, 542–3. London, 1860.

** Op. cit. 483.

†† Traite de la pneumonie. (Pneumo niesperiodiques), Deuxieme ed. Paris, 1864, 411.

‡‡ Dis. Int. Valley, N. A. 2d Series, 872.

of the sanitive effects of the quinine treatment, that they must concede that the remedy is as efficient in removing the inflammation, as it is in arresting the pyrexia.

*In Cynanche Trachealis.* In ordinary croup I have witnessed the most beneficial results from the administration of the sulphate of quinine. I was first led to its employment from having often observed its remittent character, the remissions usually occurring during the morning hours. In conjunction with tartar emetic and calomel in moderate doses, I found that quinine, freely employed, in doses of two to five grains in infantile cases, materially assisted the action of those remedies, and promptly arrested the disease in the large majority of cases. I have not lost a patient with croup since I adopted this treatment.

*In Delirium Tremens.* We have seen that it has been stated that the sulphate of quinine has induced symptoms resembling delirium tremens. That this remedy may, in large doses, enfeeble the functions of the brain, to such an extent as to give rise to symptoms resembling that disease, may be admitted as possible, although such an occurrence, in a very extended experience with the salt, has never been observed by me. Symptoms bearing a close resemblance often result from very different causes and conditions, but however this may be, in *real* delirium tremens I have seen the most prompt and decided favorable results from the exhibition of quinine. To a patient laboring under this disease, who had not slept for one hundred and twenty hours, and to whom large doses of laudanum and morphine had been given ineffectually, I gave twenty grains of the sulphate of quinine, with the effect of producing sleep in an hour after its administration. The remedy was repeated every night in smaller doses until perfect tranquillity of the innervation was established. Some of my professional friends have given the remedy with the same happy results.

*In Acute Rheumatism.* Morton* gave Peruvian bark freely in acute rheumatism, not only when affecting the intermittent type, but also when presenting a continuous character; the remedy being preceded by bleeding and purgatives. This practice was likewise followed by Saunders,† Fothergill,‡ Haygarth,§ and others. Morton administered the bark in quantities of one and two ounces with prompt relief. Saunders states that he generally began "about the seventh day from the attack with the cold infusion of the red bark in the dose of three ounces every three hours until the evening paroxysm came on." "I have found," he states, "in many cases, by this practice, the rheumatic fever greatly shortened, and

* Opera Medica, Tom. I., De prot, Feb.; int. p. 84, et De prot, febr. cont. 149.

† Obs. on the Red Peruvian Bark, London, 1783, p. 74.

‡ Med. Observ. and Inquiries, 1, 319.

§ Clinical History of Diseases, 89.

the debility and torpor in the joints, which is frequently the effect of that disease, together, with the disposition to the chronic rheumatism, generally prevented." He premised the use of the bark by anti-phlogistic treatment.

Haygarth at first gave the bark very cautiously in small doses, premising its administration by venesection and cathartics, but he afterwards gave it in quantities of one to two ounces in twelve hours, only preceded by an antimonial emetic. Under this treatment he stated "that the pains, sweats, and other symptoms of inflammatory fever manifestly and speedily abate till health is perfectly restored." It was his opinion that "except mercury in the syphilis, there are few or perhaps no examples where a remedy can produce such speedy relief and perfect recovery in so formidable a disease," and adds: "For many years I have been thoroughly convinced that the Peruvian bark has a much more powerful effect in the rheumatic than in any other fever; and that it does not even cure an ague so certainly and so quickly."

Notwithstanding these and other favorable reports of the efficacy of cinchona in rheumatism, the practice never became general, and its employment was usually limited to the last stages of the disease on account of its supposed tonic effects in relieving the exhausting sweats, etc. On the discovery of the sulphate of quinine this remedy was also tried in the disease, but did not attract much attention until M. Briquet brought it prominently forward to the notice of the profession. This physician employed the salt in large doses in rheumatism, and in his treatise gives the history of this treatment in two hundred and fifty cases of acute and chronic forms. The following extracts from his work give the result of his method—the effects on the circulation have been previously detailed:

"The sulphate of quinine, in doses of 15 to 45 and 60 grains, greatly modifies the phenomena, march and duration of acute articular rheumatism.

"It acts as a very powerful *calmant*, and soon produces restorative sleep.

"The tumefactions of the joints are notably prevented; those which are already existing are diminished or lessened in their intensity. The fever which accompanies the articular lesion is also invariably diminished in a considerable degree.

"The complications of endocarditis and pericarditis did not prevent the action of the sulphate of quinine on the principal malady, but often underwent a notable amelioration from the employment of the remedy.

"When proper precaution was taken in the choice of subjects, and in the administration of the remedy, the sulphate of quinine, even in the largest doses, did not produce any serious accident."

The experience of the writer of this essay, in the employment of the sulphate of quinine in acute rheumatism, although not very extensive, has been highly satisfactory. I have not, however, relied upon it solely. In plethoric subjects, the action of the remedy has always been assisted by the abstraction of blood, and in other cases by the administration of colchicum, aperients, etc. I have always found the pulse diminished in force, volume and frequency, under its use, and the violence and march of the disease controlled in an evident degree. I have seen no exasperation of the disease, or its complications, from its employment in any case. When we reflect upon the usual tedious duration of acute rheumatism, the agonizing suffering attendant upon it, and the serious organic lesions to which it so often gives rise, we should welcome any remedy which would diminish its violence and abridge its duration. That the sulphate of quinine has produced these salutary results there can be no reasonable doubt, and I feel assured that it will eventually be considered one of the most valuable additions to the therapeutics of the disease.

*In Chronic Rheumatism.* The effects of the sulphate of quinine in the treatment of chronic rheumatism, though not so immediate and decisive, are generally conceded to be very beneficial. I have nothing to add on this subject, to the information to be found in works generally accessible.

*In Cholera Infantum.* I have witnessed the most beneficial effects from the employment of the sulphate of quinine in this disease. I was first led to use it in this fatal malady from observing the unequal distribution of animal heat to be discerned in the majority of cases, and the strong tendency to congestion of the brain, in the worst grades of the affection. Given in sedative doses, I have not only seen these symptoms promptly removed, but also have generally observed the vomiting and purging to cease. I have seen it rescue cases in the last stages of this malady, when the extremities were cold and the patient in profound coma. Did the limits of this paper admit, I could relate almost miraculous recoveries from the use of the remedy in this disease, in apparently hopeless conditions.

In cholera infantum I usually prescribe it in doses of 1 to 3 grains (carefully watching its effects lest its sedative action might transcend the desirable degree) and repeating the dose every two or three hours, according to circumstances. In the chronic diarrhœa following attacks of cholera infantum, I usually employ it in small doses, in combination with tannin and subnitrate of bismuth.—*Virginia Clinical Record.*

